# Mycotic Abdominal Aortic Aneurysm in the Endovascular Era

**DOI:** 10.7759/cureus.6119

**Published:** 2019-11-11

**Authors:** Ian P Barry

**Affiliations:** 1 Vascular and Endovascular Surgery, Fiona Stanley Hospital, Perth, AUS

**Keywords:** mycotic aneurysm, aneurysm, salmonella, aortitis, endovascular aneurysm repair (evar), evar, pseudo-aneurysm, aortic aneurysm

## Abstract

A mycotic aneurysm is a localised dilatation of an artery due to destruction of the vessel wall by infection. Diagnosis is based on clinical, microbiological, and radiological findings. Typical management includes antibiotic therapy and open surgical debridement with or without revascularisation. This case of mycotic aortic aneurysm highlights the utilisation of endovascular grafts in the treatment of such pathology. This may improve both short and long term morbidity and mortality as compared to open intervention.

## Introduction

A mycotic aneurysm is a localised dilatation of an artery due to destruction of the vessel wall by infection [1]. It may be classified as true (involving all three layers of the arterial wall) or false (collection of blood that forms between the two outer layers of an artery) [1]. It may occur due to a pre-existing aneurysm becoming secondarily infected or infection of the arterial wall resulting in the development of a new aneurysm. Risk factors include antecedent infection, arterial injury, and impaired immunity [2-4]. In the setting of local arterial wall infection, localised perforation and pseudo aneurysm may result.

The organisms with the greatest affinity for the aortic wall are Staphylococcus and Salmonella spp. [5]. Salmonella is less prevalent in comparison but has been reported in up to 15% of cases with bacteremic seeding of atherosclerotic plaque proposed [6-7]. 

Typical management includes antibiotic therapy and open surgical debridement with or without revascularisation [8]. However, endovascular techniques are now being utilised as a treatment alternative to avoid the high morbidity and mortality associated with open aortic surgery.

## Case presentation

An 82-year-old male presented to his local hospital with mild chest pain whereby he was given a diagnosis of pericarditis and discharged home. At this stage, discomfort predominantly affected his lower abdomen and lumbar spine with minimal radiation to the medial thigh. Ongoing subjective fevers, lethargy, and nausea led to re-presentation to his local emergency department. Nil other infective symptoms were highlighted with the patient denying any recent cough, dysuria, or diarrhoea.

On examination, he was noted to be tachycardic at 110 bpm with a temperature of 38.5 degrees (Celsius). Abdominal palpation revealed suprapubic tenderness without evidence of peritonism. No arterial compromise was identified to the bilateral lower limbs.

**Figure 1:**
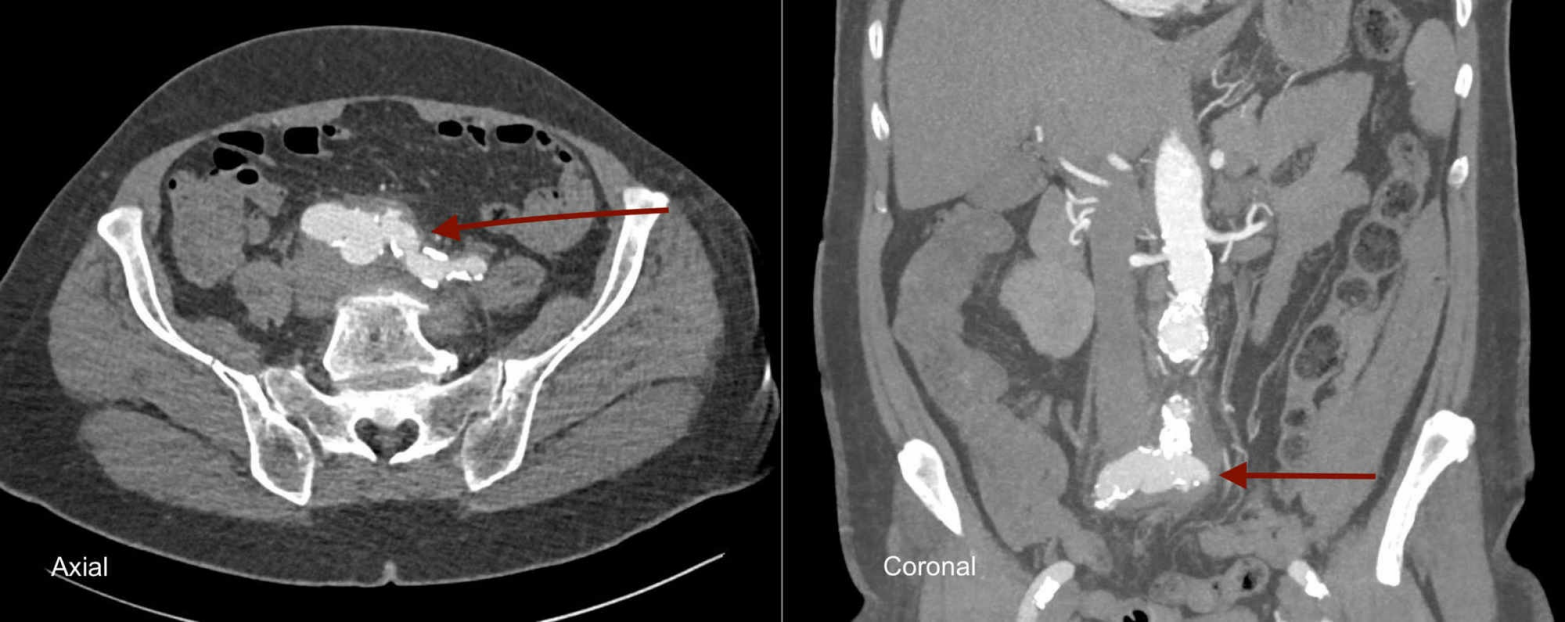
Mycotic aneurysm of aortic bifurcation (axial and coronal contrast-enhanced computed tomography)

Laboratory investigations revealed a white cell count of 12.27 x 10^9/L (normal range 4-11 x 10^9/L) with a c-reactive protein (CRP) of 240 mg/L (normal range <5 mg/L). Blood cultures were sent and broad-spectrum antibiotics were commenced (as per local hospital guidelines). Urgent contrast-enhanced computed tomography (CT) of the abdomen and pelvis was performed. This highlighted the aneurysmal dilatation of the aortic bifurcation extending into both common iliac arteries with retroperitoneal inflammatory fat-stranding (Figure 1).

He was emergently transferred to a tertiary hospital where he could receive specialist care. Blood cultures grew Salmonella spp. with the subsequent rationalisation of his antibiotic regimen. A multi-disciplinary approach was utilised in the development of an appropriate management strategy with vascular surgery, microbiology, and infectious diseases involved. In light of the anatomical considerations, as well as the high morbidity and mortality associated with open surgical repair, endovascular intervention with lifelong antibiotic therapy was proposed.

**Figure 2:**
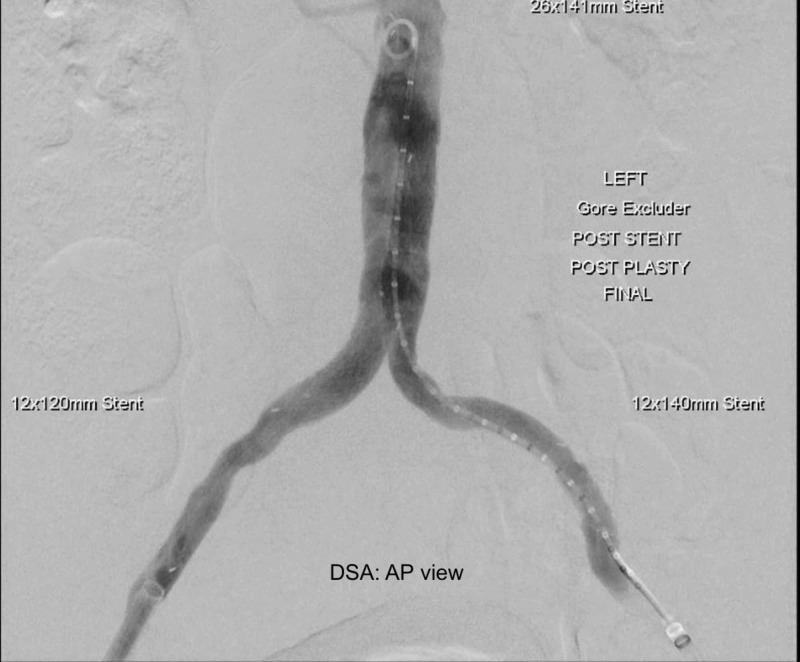
Completion angiogram following endovascular aneurysm repair (EVAR)

The patient consented to endovascular aortic aneurysm repair (EVAR) with a Gore Excluder infra-renal stent (WL Gore and Associates, Flagstaff, AZ) (26 x 14 x 140 mm) and bilateral internal iliac artery (IIA) embolization via amplatzer plugs (9 x 16 mm and 9 x 14 mm). Embolisation of the IIA's was undertaken to ensure an adequate seal and to avoid the development of an endoleak which was absent on the completion angiogram (Figure 2).

Following this, he returned to the ward and had an uncomplicated post-operative course. He was discharged home on day three following EVAR with ongoing intravenous antibiotics and specialist infectious disease and vascular follow-up arranged.

## Discussion

Abdominal aortic aneurysms of non-infective or non-inflammatory origin have been managed via endovascular means, where appropriate, for many years (imaging plays an essential role in the delineation of these pathologies with this case highlighting several mycotic features including multi-lobulated appearance and peri-aortic inflammation). However, the utilisation of such techniques in mycotic aortic aneurysm is in its infancy. There are no randomised control trials to guide the management of such aneurysms with strategies predominantly based upon clinical experience and case series.

Endovascular therapy may therefore offer an alternative treatment modality, specifically for those where co-morbidity precludes open aortic surgery. This was highlighted by Kan et al. with a 90% 30-day survival and an 82% two-year survival identified in those who underwent endovascular repair for mycotic thoracic or abdominal aortic aneurysms [9]. A Swedish study also highlighted significantly reduced perioperative mortality in those treated endovascularly as compared to open surgical repair (three-month survival; 96% versus 74%) with comparable long term outcomes [10].

A mycotic aortic aneurysm is a life-threatening condition and the ongoing development of endovascular grafts aligned to the ever increasing surgical expertise may improve both short and long term outcomes. This case highlights the low peri-operative morbidity/mortality associated with such interventions.

## Conclusions

Salmonella aortitis/mycotic aneurysm is a rare condition which may rapidly progress to pseudo aneurysm formation and rupture. It is important to recognise this condition so prompt treatment may be initiated. Diagnosis is based on clinical, microbiological, and radiological findings. Optimal treatment strategies require further investigation. This case demonstrates the symptoms and signs, aetiology, and radiological findings of a mycotic abdominal aortic aneurysm as well as the effective utilisation of endovascular techniques in acute management.
